# Multiplexed high-throughput immune cell imaging reveals molecular health-associated phenotypes

**DOI:** 10.1126/sciadv.abn5631

**Published:** 2022-11-02

**Authors:** Yannik Severin, Benjamin D. Hale, Julien Mena, David Goslings, Beat M. Frey, Berend Snijder

**Affiliations:** ^1^ Department of Biology, Institute of Molecular Systems Biology, ETH Zürich, 8049 Zürich, Switzerland.; ^2^ Blood Transfusion Service Zürich, SRC, 8952 Schlieren, Switzerland.

## Abstract

Phenotypic plasticity is essential to the immune system, yet the factors that shape it are not fully understood. Here, we comprehensively analyze immune cell phenotypes including morphology across human cohorts by single-round multiplexed immunofluorescence, automated microscopy, and deep learning. Using the uncertainty of convolutional neural networks to cluster the phenotypes of eight distinct immune cell subsets, we find that the resulting maps are influenced by donor age, gender, and blood pressure, revealing distinct polarization and activation-associated phenotypes across immune cell classes. We further associate T cell morphology to transcriptional state based on their joint donor variability and validate an inflammation-associated polarized T cell morphology and an age-associated loss of mitochondria in CD4^+^ T cells. Together, we show that immune cell phenotypes reflect both molecular and personal health information, opening new perspectives into the deep immune phenotyping of individual people in health and disease.

## Teaser

Imaging human immune cells reveals morphologies that reflect health information of the donor, such as age and inflammation.

## INTRODUCTION

The morphology of a cell closely reflects its state, as it adapts to dynamic functional requirements and thereby constrains future behavior (*1*–*4*). This feedback mechanism has been shown to influence many cellular events, including cell differentiation (*5*, *6*), cell division (*2*, *7*, *8*), adaptation to the microenvironment (*9*–*11*), and malignant transformation (*12*, *13*). Few differentiated healthy human cells change their phenotype as markedly as immune cells: a plasticity that is critical to the correct function of the immune system as a whole (*14*–*16*). As a consequence, studying immune cellular heterogeneity at the molecular level has been transformative for our understanding of the immune system, measured, for example, by flow cytometry (*17*, *18*), single-cell mass cytometry (*19*, *20*), and single-cell RNA sequencing (RNA-seq) (*21*–*25*). Complementary to these molecular measurements, microscopy has shown the importance of immune cell morphology in multiple settings: Distinct cellular morphologies are associated with, and influence the outcome of, monocyte polarization (*26*, *27*) and T and B cell activation (*28*–*33*), and label-free imaging of hematopoietic cells has enabled predicting the outcome of future lineage choices (*34*). In addition, a recent study, using organelle marker abundance as a proxy for cell morphology, found extensive evidence for morphological heterogeneity in both healthy and diseased immune cells (*35*). Because of their mixed adherent nature, however, primary immune cells such as peripheral blood mononuclear cells (PBMCs) were long considered incompatible with automated fluorescence microscopy, the tool of choice to characterize cellular morphology with spatial resolution across millions of cells (*4*, *9*, *11*, *36*–*39*). This has hampered the comprehensive measurement and study of morphological heterogeneity present in the immune system and thus has left unanswered the question of which molecular and health factors globally shape the compendium of human immune cell morphologies.

## RESULTS

### Deep learning enables accurate eight-class cell type classification on multiplexed immunofluorescence and automated microscopy of human immune cells

**Fig. 1.**
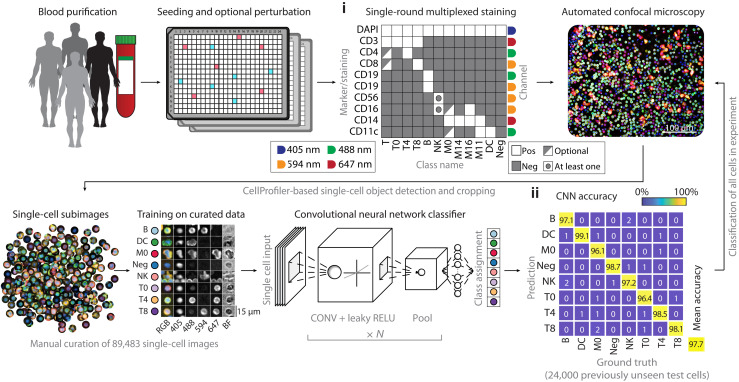
Deep learning–enabled multiplexed immunofluorescence and microscopy of human immune cells. Workflow for the single-round multiplexed immunofluorescence, image-based screening, and associated deep learning–based classification of human PBMCs. PBMCs of healthy human donors are seeded in 384-well plates, optionally containing drugs or immune stimuli. Cells are fixed and stained with a comprehensive antibody panel (i) and imaged by automated confocal microscopy. A CNN is trained on 89,483 manually curated subimages to distinguish eight different immune cell classes and subsequently classifies all cells in the experiment. The curated test set contains 100 cells per class per donor per staining condition. (ii) Confusion matrix of CNN performance across all 24,000 cells that the CNN did not see before. CONV, convolution; RELU, rectified linear unit.

To be able to comprehensively measure immune cell phenotypes, we developed a multiplexed immunofluorescence approach for PBMCs that extends our previously developed protocol for high-throughput image-based screening in human biopsies compatible with mixed nonadherent cells (Fig. 1) (*40*–*42*). In contrast to previously reported cyclical multiplexed immunofluorescence protocols (*43*–*45*), we stain once with a comprehensive immune cell marker panel that multiplexes eight surface markers and a nuclear dye, which is imaged by automated confocal microscopy and bright-field imaging in a single run (Fig. 1i and table S1). A deep convolutional neural network (CNN) (*46*) with custom architecture (fig. S1A) was subsequently used to classify each cell, making use of distinct marker expression patterns, lineage-specific labeling encoded by the staining panel, and likely differences in immune cell morphology (Fig. 1i). The CNN was trained across eight immune cell classes, using 89,483 manually curated five-channel subimages (four fluorescent channels and one bright-field channel) centered on individual cells sampled from 15 healthy donors (available at https://doi.org/10.3929/ethz-b-000343106). The eight immune classes capture the predominant immune lineages present in PBMCs, including three distinct T cell subsets (CD4^+^, CD8^+^, and CD4^−^CD8^−^), monocytes, dendritic cells, natural killer (NK) cells, B cells, and nucleated immune cells negative for all eight surface markers (Fig. 1).

CNN performance was stable across retraining, showed no sign of overfitting, and was 97% accurate for unseen donors systematically left out of the training data (fig. S1, B and C). The network further achieved 97.7% classification accuracy (Fig. 1ii) on a previously unseen test dataset of 24,000 curated cells comprising PBMCs from the same 15 healthy donors (fig. S1D). The classification efficiently demultiplexed mixed marker signals in the same fluorescence channel (fig. S1E), such that the resulting abundances of each subpopulation matched our expectations (fig. S1F). Both the class fractions (fig. S1G) and class probabilities (fig. S1H) showed good reproducibility over different experimental replicates [median correlation coefficient (*r*) = 0.90 and 0.95, respectively]. While marker expression likely contributed toward the accurate classification of morphologically similar classes (such as CD4^+^ and CD8^+^ T cells), cell morphology likely contributed to the separation of distinct cell types whose markers were multiplexed in the same channel. For example, CD14^+^ monocytes and CD3^+^ T cells, both stained in the allophycocyanin (APC) channel (fig. S1E), showed separation in cell size and staining pattern (fig. S1I). Supporting this interpretation, retraining the eight-class CNN without 4′,6-diamidino-2-phenylindole (DAPI) and bright-field channels significantly reduced the classification accuracy (*P* < 0.05; fig. S1J); in addition, inversely, a two-class CNN could separate T cells and monocytes with 95% accuracy based on the DAPI and bright-field channels alone (fig. S1K). Last, we evaluated withholding all morphological information from a neural network classifier, by training an eight-class fully connected neural network classifier on the mean channel intensities per cell. This resulted in a classification accuracy of 86.2%, considerably lower than that of the CNN (fig. S1L). Thus, the eight-class CNN learned to generalize and leverage immune phenotypes across individual donors and experiments, presenting a robust, efficient, and data-rich high-throughput screening strategy with broad applicability.

### Neural network uncertainty clusters immune cell phenotypes

**Fig. 2.**
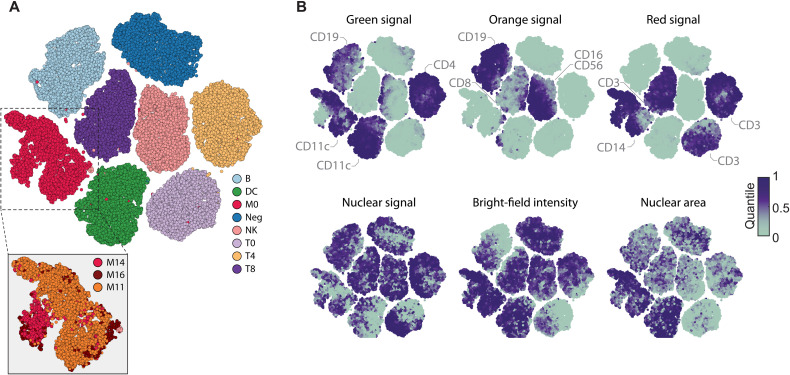
Phenotypic landscape of the unperturbed immune system across 10 healthy donors. (**A**) *t*-SNE of the eight-class CNN probabilities of up to 1000 randomly subsampled high-confidence multiplexed cells per class and per donor (class probability > 0.7). Monocytes are further divided into three subpopulations by thresholding the immunofluorescence (IF) intensity of CD16 and CD11c stainings, respectively (inset). Figure depicts a total of 78,850 cells, randomly sampled from 40 wells for each of the 10 donors. All donors were processed and measured together in a single experiment, across 40 replicate wells per donor distributed over two 384-well plates. (**B**) Selected single-cell features projected onto the *t*-SNE shown in (A). Median value of overlapping data points is calculated, and color is assigned accordingly. Points are plotted in order of intensity, with the lowest intensity on top.

Both supervised and unsupervised deep learning algorithms are increasingly used for image clustering (*47*, *48*), which we here explored for the purpose of clustering immune cell phenotypes. The CNN returns a confidence vector for each cell that creates an eight-dimensional feature space, which we visualized by *t*-distributed stochastic neighbor embedding (*t*-SNE) (Fig. 2A) (*49*). To minimize possible batch effects and confounding factors from ex vivo culturing, we analyzed a subset of 10 of the 15 donors on which the CNN was trained, whose blood had been simultaneously processed and incubated for just 1 hour before fixing and imaging across replicate wells and plates. Visualization of unperturbed immune cells from these 10 donors suggested considerable cell-to-cell variability, particularly among monocytes, even just within the cells classified with high CNN confidence (>0.7 class probability) (Fig. 2A). Projecting molecular and morphological cell features measured by conventional image analysis on the *t-*SNE revealed that the CNN had separated monocytes on the basis of their CD16 and CD11c expression levels, although it was not trained explicitly to do so (Fig. 2A, inset). Moreover, this showed that, even for high-confidence cells, the CNN class probabilities reflected marker expression and morphological heterogeneity for all eight immune cell classes, with nuclear size and bright-field intensity differences observed within each class (Fig. 2, A and B). Thus, while the eight-class CNN was strictly trained in a supervised manner, its neural network uncertainty additionally allowed further grouping of previously unannotated cellular phenotypes, capturing recurrent phenotypes present in primary human immune cells.

We next tested whether this deep learning uncertainty could also be used to quantify and categorize extrinsically induced changes in immune cell phenotypes. To this end, we stimulated PBMCs with nine immune modulators and three distinct controls ex vivo across concentrations and replicates, measuring 5 million multiplexed stained and imaged PBMCs (table S2). To avoid a possible bias from functional differences between immune cells from different donors, all cells were sampled from a single additional donor.

**Fig. 3.**
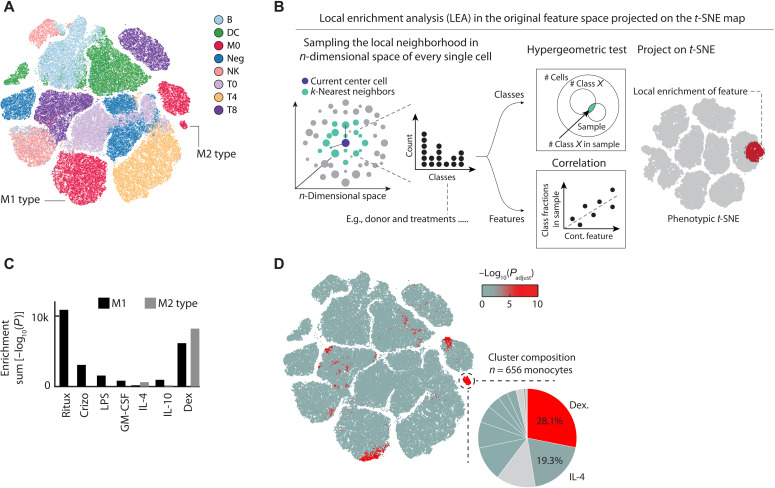
Phenotypic landscape of a perturbed immune system and LEA analysis. (**A**) The CNN class probability *t*-SNE on the left shows 600 randomly chosen cells per class and condition; colored by class. (**B**) Overview of the LEA workflow. LEA probes the KNNs of each single cell in a multidimensional space for enrichment of either continuous or discrete features. For discrete features, the probability of finding *n* cells of condition *X* in the probed neighborhood follows a hypergeometric distribution, from which an enrichment *P* value is calculated. For continuous features, the relative fraction of cells of each donor in the probed local neighborhood is calculated. These fractions are then rank-correlated with a continuous feature that was measured across donors, and the *P* value of the correlation is calculated. In both cases, the enrichment probability is assigned to the center cell, and the approach is iterated for each single cell in the analysis. (**C**) Bar graph depicting the sum total log_10_(LEA *P* values) for selected perturbations in the M1 (black) and M2 (gray) monocyte clusters. (**D**) LEA reveals regions in the phenotypic space that are significantly enriched for dexamethasone-treated PBMCs. Cells in the *t*-SNE are colored by their enrichment significance of the LEA run [−log_10_(*P*_adjust_); see color bar]. Inset highlights the contribution of different perturbations to the selected M2-type monocyte cluster. Figure depicts a total of 199,375 cells, randomly sampled from across 240 wells for a single donor.

First, we visualized the structure in the CNN confidence by *t*-SNE (Fig. 3A), equally sampling cells from across all eight classes and 12 conditions. This revealed monocytes to be divided into three clusters associated with distinct CNN confidence profiles, not trivially explained by marker expression differences (fig. S2, A to C). To identify the contribution of distinct immune modulators to the morphological landscape of immune cells, we developed a method called *k*-nearest neighbor (KNN) local enrichment analysis (LEA) by hypergeometric testing (Fig. 3B and Material and Methods). For each cell, LEA identifies the nearest neighbors in the original eight-class probability space and calculates the hypergeometric significance of enrichment for cells with a certain property in this neighborhood. LEA next assigns this significance back to the original starting cell (see data file S1 for an example of this analysis). Projecting the LEA results back on the *t*-SNE revealed that the monocyte subcluster with the lowest CNN confidence was enriched for monocytes exposed to M1-type–inducing agents, *Escherichia coli* lipopolysaccharides (LPSs) and granulocyte-macrophage colony-stimulating factor (GM-CSF) (Fig. 3C and fig. S2D) (*50*), or to cytotoxic agents, causing the release of danger-associated molecular patterns. The second monocyte cluster was strongly enriched for cells exposed to M2-type–associated dexamethasone or interleukin-4 (IL-4), while the third and highest-confidence monocyte cluster was not enriched for most perturbations, thus likely reflecting unperturbed monocyte phenotypes (Fig. 3D).

Stimulation with microbial compounds such as LPS can selectively alter immune cell cross-talk, for example, through the induction of cell-cell contacts. We therefore suspected that phenotypes in the M1-type cluster could, in part, reflect changes in the multicellular context. To verify this, we performed spatially resolved single-cell analysis across the eight classified immune cell types, allowing the high-throughput screening of 36 distinct immune cell-cell interactions simultaneously, a significant increase compared to our previous nonmultiplexed efforts (fig. S3, A and B) (*40*). Analysis of all 43 million cell-cell interactions measured in this experiment (fig. S3A) confirmed the M1-like monocyte cluster to be enriched for monocyte-to-monocyte interactions (fig. S3C). Thus, LPS-mediated monocyte activation led to distinct M1-like monocyte phenotypes, defined, in part, by an altered multicellular context. Collectively, LEA revealed that the uncertainty of the deep neural network reconstituted previously established monocyte M1/M2-type polarization phenotypes in a fully unsupervised manner (Fig. 3), while exposing considerably phenotypic complexity, with most immunomodulatory perturbations simultaneously affecting the phenotype of multiple immune cell class (Fig. 3A and fig. S2D).

### Immune cell phenotypes associate with personal health information

**Fig. 4.**
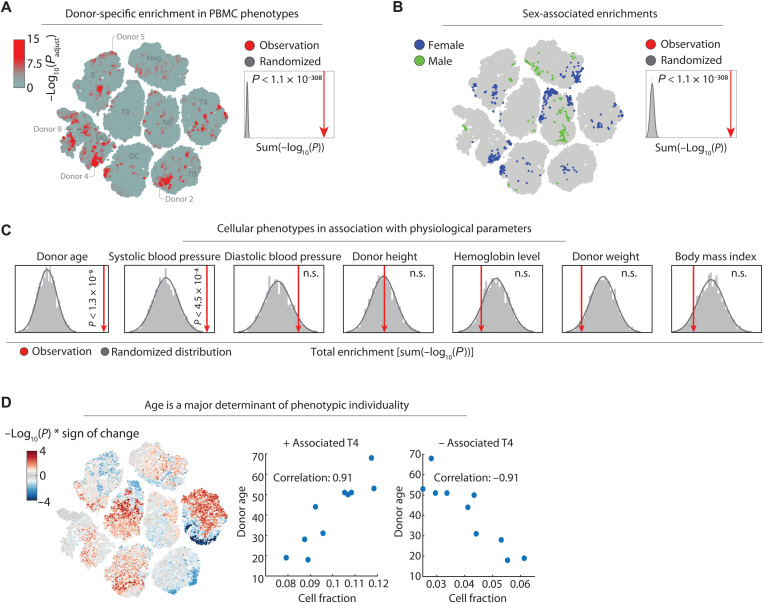
LEA associates immune cell phenotypes with personal health information. (**A**) LEA of donor-specific cells across 10 donors, visualized on the *t*-SNE of Fig. 2A. The cells are colored by their maximum LEA significance across the 10 donors [−log_10_(*P*_adjust_); see color bar). Inset: A randomized null distribution of donor enrichments was generated by randomizing the donor labels 2000 times and summing up all single-cell enrichments calculated by LEA per randomized run (gray bars). Sum enrichment of the actual data is shown in red, and the significance compared to the randomized runs is calculated by a one-sided *t* test. (**B**) LEA of biological gender-specific phenotypes projected onto the *t*-SNE. Cells are colored by their significant enrichment in female (blue) or male (green) specific phenotypes. Inset: A null distribution of random gender enrichment (gray) was generated by randomizing the donor labels 2000 times and summing up all single-cell enrichments calculated by LEA. Sum enrichment of the actual data is shown in red, as in Fig. 3A. (**C**) Association analysis of various health parameters with cellular phenotypes calculated by LEA. Null distributions of random correlation significance (gray) were generated by randomizing the donor labels 2000 times and summing up the all single-cell enrichments calculated by LEA per randomized run. Enrichment of the actual data is shown in red (one-sided *t* test). n.s., not significant. (**D**) LEA age associations projected onto the *t*-SNE. Single cells are colored by their signed significance of correlation [−log_10_(*P*) * sign of the correlation; see color bar]. Inset: Fraction of all significantly positive and negative age-associated CD4^+^ T cells with donor age (*P* < 0.05).

The phenotypic heterogeneity of circulating immune cells captured by our image-based measurements could reflect both genetic and nongenetic influences (*15*, *51*). To explore this, we analyzed commonalities and differences in the unperturbed immune phenotypes across the discovery cohort of the 10 donors shown in Fig. 2. We first used LEA to measure enrichment of cells from the same donor in the nearest-neighborhood in the eight-dimensional CNN class probability space. This identified distinct cellular phenotype regions significantly enriched for each of the 10 donors across several immune cell classes (Fig. 4A). As these enriched phenotypes were measured across technical repeats, they not only potentially indicated donor-individual characteristics of immune cell morphologies but could also reflect batch effects acting upstream of our sample processing and imaging. Repeating the analysis with randomized donor labels and comparing the sum of enrichments showed that the actual donor enrichment in nearest neighbors of the latent space was well above what would be expected by random (*P* < 1.1 × 10^−308^; Fig. 4A, inset). We next looked for phenotypes that were enriched in donors with the same biological gender, with the 10 donors including 4 women and 6 men. This revealed strong gender associations with various immune cell morphologies (*P* < 1.1 × 10^−308^; Fig. 4B), with NK and negative cell class phenotypes particularly enriched in female donors and not explained by enrichment in any individual female donor (fig. S4A).

We next explored immune phenotype associations with continuous health parameters such as donor age, which has been described to markedly alter the immune phenotypic landscape (Figs. 3B and 4C and Material and Methods) (*52*, *53*). A modification of LEA for continuous variables calculates the significance of the rank correlation between the fraction of cells per donor in the nearest neighborhood and any continuous variable of each donor (Fig. 3B). As before, the LEA analysis was run in the eight-dimensional CNN class probability space. To correct for spurious associations, we compare the association strength with those observed in many repeats with the same health parameter randomized across the donors. Testing donor age, height, weight, body mass index, blood pressure, and hemoglobin levels revealed significant associations with donor age (*P* < 1.3 × 10^−9^) and systolic blood pressure (*P* < 4.5 × 10^−4^; Fig. 4C) but not to any of the other measured health parameters. The age-associated phenotype map revealed bimodal age associations for several immune subpopulations, particularly notable for CD4^+^ T cells (Fig. 4D). Across the cells that make up the phenotype map, the age associations were mutually exclusive of the single-donor enrichments (*r* = −0.002; fig. S4B).

### Donor variability allows to link T cell phenotypes with bulk gene expression data

**Fig. 5.**
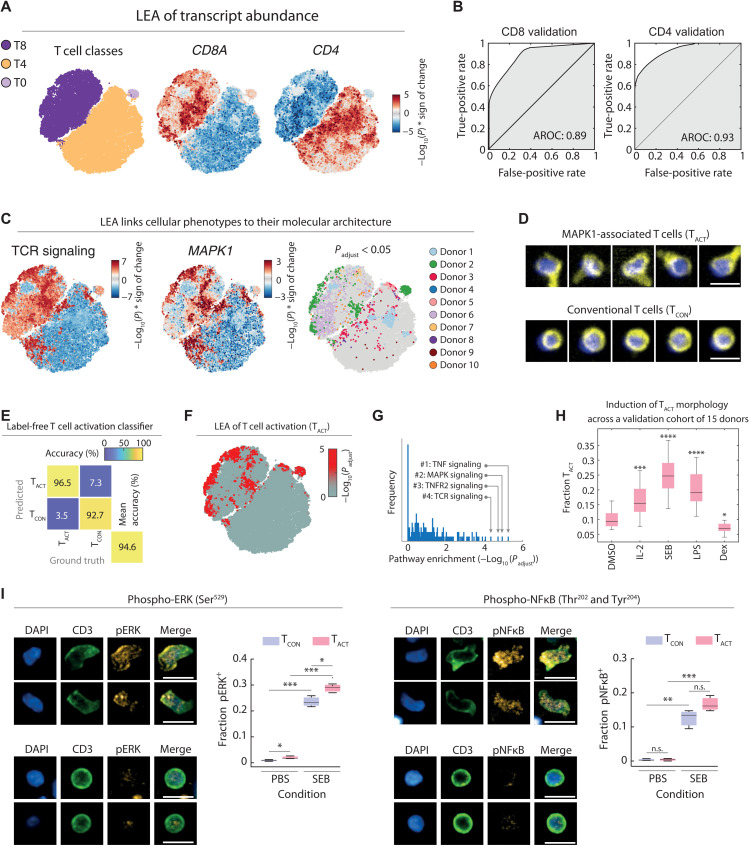
Discovery and validation of an activated T cell morphology. (**A**) *t*-SNE of the CNN class probabilities of T cells from 10 healthy donors. A total of 10,000 T cells were randomly selected across all T cell populations per donor (without confidence threshold) to reflect their original abundance in the respective sample. *t*-SNE colored per T cell class (left), by LEA-based associations with *CD8A* transcript abundance (middle), or *CD4* transcript abundance (right) [−log_10_(*P*) * correlation sign]. (**B**) Area under the receiver operating characteristic curve (AROC) for the consistency between the *CD8A* LEA associations with CD8 levels by immunofluorescence (left) and the *CD4* LEA associations with CD4 levels by immunofluorescence (right). (**C**) LEA associations of TCR signaling (left), *MAPK1* transcript abundance (middle), and donor enriched regions (right; *P*_adjust_ < 0.05 colored per donor) projected on the *t*-SNE map. (**D**) Representative CD8^+^ T cells from the positively MAPK1-associated regions (T_ACT_) and conventional CD8^+^ T cells of other regions (T_CON_). Yellow, CD3; blue, DAPI. (**E**) Confusion matrix of the T_ACT_ classifier. Test set comprises 369 random T_ACT_ cells and 738 random T_CON_ cells across multiple donors (including the 10 depicted donors). (**F**) LEA of the T_ACT_ phenotype projected on the *t*-SNE map. (**G**) Distribution of pathway significance across all retroactively classified T_ACT_ morphologies. Pathway enrichments were calculated using a hypergeometric test on positively associated genes (0.95 percentile), and *P* values were corrected for multiple testing. (**H**) Induction and suppression of the T_ACT_ phenotype with immunomodulatory agents across an independent validation cohort of 15 individual donors. Compounds were screened at 100 ng/ml. Box plots show the mean relative fraction of T_ACT_ of all T cells across all wells of each condition per donor. (**I**) Immunofluorescence quantification of phospho–nuclear factor κB (pNFκB) and phospho–extracellular signal–regulated kinase (pERK) levels in T_ACT_ and T_CON_ cells. Box plots show the fraction of phospho-signaling positive T_ACT_ (red) and T_CON_ (blue) cells after 48 hours of incubation with SEB or control (*n* = 3). Representative T_ACT_ and T_CON_ cell morphologies shown. Scale bars, 10 μm. *P < 0.05, **P < 0.01, ***P < 0.001, ****P < 0.0001.

To investigate the above identified phenotypic and health associations, we next used LEA to associate molecular pathway expression as measured by transcriptomics with immune cell phenotypes. Focusing on T cells, we performed bulk RNA-seq of CD3-positive cells isolated from the same 10 healthy donor blood samples, detecting on average around 15,000 expressed transcripts (fig. S5A). To associate bulk transcript measurements with single-cell imaging data, we first randomly subsampled 10,000 imaged T cells per donor, irrespective of their subpopulations (T0, T4, and T8). This random subsampling was performed to reflect the composition of isolated bulk T cells on which RNA-seq was performed. We then correlated the local phenotype abundance in the eight-dimensional CNN class probability space with the bulk transcript measurement using LEA across donors (Fig. 5A). To benchmark these phenotype-to-transcriptome associations, we first compared the LEA associations of *CD4* and *CD8A* transcript abundance (Fig. 5A) with the CD4 and CD8 protein expression levels explicitly measured by immunofluorescence for each T cell (fig. S5B). Validating the approach, LEA achieved excellent results for these proof-of-concept benchmarks, with areas under the receiver operating curve of 0.93 and 0.89 for CD4- and CD8-positive cells, respectively (Fig. 5B).

We next sought to validate these pathway-phenotype associations by querying the associations the other way round: starting from well-known pathways and seeing what phenotypes are associated with it. To this end, we inspected the associations with the T cell receptor (TCR) signaling pathway as proxy for T cell activation. TCR signaling was strongly associated with distinct subregions of the phenotype map, including the cluster periphery of CD8^+^ T cells (Fig. 5C). This pattern was recapitulated by the LEA associations with *MAPK1* (*ERK2*), part of the TCR-induced signaling cascade, which largely, but not exclusively, overlapped with regions enriched for cells from donor 2 (Fig. 5C). Visual inspection of cells residing in TCR signaling and *MAPK1-*associated phenotypic regions revealed a notable polarized and activated T cell morphology, henceforth referred to as T_ACT_ cells. In contrast, randomly sampled cells from adjacent and nonenriched regions contained conventional small and round T cell morphologies, which we refer to as T_CON_ cells (Fig. 5D). To robustly quantify the T_ACT_ morphology further, we trained a dedicated CNN on manually curated T_ACT_ and T_CON_ phenotypes, which achieved 94.6% validation accuracy on images from donors and experiments that it was not trained on (Fig. 5E and fig. S5C). This allowed us to retroactively detect the T_ACT_ morphology for all imaged T cells, which confirmed that the phenotype was present in all donors and most enriched in the cells of donor 2 (Fig. 5F and fig. S5D). Coming full circle, the T_ACT_ enriched regions associated with tumor necrosis factor (TNF) and mitogen-activated protein kinase (MAPK) signaling as most enriched pathways after multiple testing correction (Fig. 5G).

### Validation of the inflammation-associated T cell morphology (T_ACT_) in an independent donor cohort

To confirm that the T_ACT_ morphology is associated with inflammation and T cell activation in an independent validation cohort, we stimulated PBMCs derived from 15 additional healthy donors with proinflammatory cytokine IL-2, superantigen *Staphylococcus aureus* enterotoxin B (SEB), or LPS, which all led to significant increases in the fraction of T cells adopting a T_ACT_ morphology (Fig. 5H and fig. S5E). Exposure to the anti-inflammatory synthetic glucocorticoid dexamethasone, in contrast, reduced the relative abundance of T_ACT_ cells across the 15 donors (Fig. 5H and fig. S5E). To rule out the possibility that the T_ACT_ morphology was induced by cellular fixation before imaging, we further conducted live cell imaging of SEB-stimulated PBMCs and visually confirmed the induction of the T_ACT_ cell phenotype (fig. S5F). We next measured by immunofluorescence the levels of phosphorylated nuclear factor κB (NFκB) (Ser^529^) and extracellular signal–regulated kinase (ERK) (Thr^202^ and Tyr^204^) as a function of T cell morphology, at baseline and upon SEB stimulation in PBMCs. At baseline, T_ACT_ cells showed slightly but significantly higher levels of phosphorylated ERK. SEB stimulation increased phosphorylated levels of ERK significantly higher in T_ACT_ than T_CON_ cells. Together, these results experimentally validated the LEA-based pathway enrichment analysis with the polarized T_ACT_ morphology. Thus, part of the donor unique fingerprints that we previously observed had resulted from differences in T cell activation between the donors, with 15% of T cells from donor 2 adopting the T_ACT_ morphology, predominantly in CD8^+^ T cell compartment, while on the other end of the spectrum, only 7% of donor 1 T cells were T_ACT_ cells, here mostly in CD4^+^ T cells (fig. S5D).

### Deep learning uncertainty reveals an age-associated mitochondrial decline in CD4^+^ T cells

**Fig. 6.**
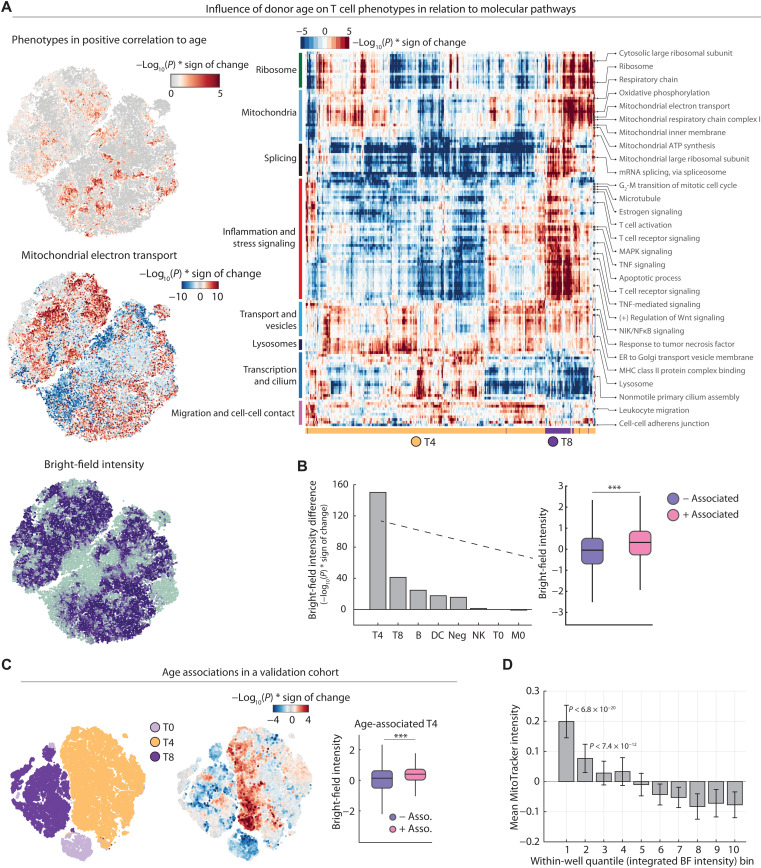
Discovery and validation of an age-associated mitochondrial decline in CD4^+^ T cells. (**A**) Top left: Positive LEA associations with donor age projected on the *t*-SNE as in Fig. 5A [colored by −log_10_(*P*)]. Middle left: LEA associations of mitochondrial electron transport projected on the *t*-SNE. Bottom left: Bright-field single-cell intensity projected onto the *t*-SNE. Median value of overlapping data points is calculated, and color is assigned accordingly. Points are plotted in order of intensity, with the lowest intensity on top. Right: Heatmap overview of all significantly enriched pathways in positive age-associated T cells [−log_10_(*P*) > 5]. ATP, adenosine 5′-triphosphate; NIK, NFκB–inducing kinase; ER, endoplasmic reticulum; MHC, major histocompatibility complex. (**B**) Comparison of the significance in difference in bright-field intensity of positively versus negatively associated immune cells per population [with an association cutoff of −log_10_(*P*) > 1.3]. Bar plots show the −log_10_(*P*) * sign of the change {1 – [median(positive enrichment) / median (negative enrichment)]}. (**C**) Negative and positive age associations with cellular T cell phenotypes and donor age in an independent validation cohort of 15 healthy individuals calculated by LEA (left and middle). *t*-SNE depicts a total of 5000 cells per donor. Right: Comparison of differences in bright-field intensity of positively versus negatively age-associated CD4^+^ T cells [with an association cutoff of −log_10_(*P*) > 1.3]. (**D**) Mitochondrial content (as measured by MitoTracker) of CD4^+^ T cells decreases with increased bright-field (BF) intensity. Bar plots display the mean MitoTracker intensity of CD4^−^ T cells per well per 10-percentile bins of bright-field intensity within each well. Mean and SDs across 10 repeat wells with a combined total of *n* = 78,095 CD4^+^ T cells are shown. *P* values are from a two-tailed *t* test of all replicate wells per bin against those of the brightest bright-field (right most) bin. ***P < 0.001.

Having validated the phenotype-to-pathway association approach and its ability to find and correctly describe new cellular phenotypes, we explored the pathway enrichments for age-associated T cell phenotypes (Fig. 6A and fig. S6A). Pathways enriched in phenotypes that were reduced with age included nucleotide excision repair, telomere maintenance (*54*), cilia assembly (*55*), and propanoate metabolism (fig. S6A). In contrast, pathways associated with T cell phenotypes that increased with age included inflammation- and stress-related pathways, particularly for the CD8^+^ compartment, and lysosome- and vesicle-associated pathways in CD4^+^ T cells (Fig. 6A, right). Inflammation is a well-described risk factor for age-associated diseases (*56*), and, consistently, the age-associated phenotypes overlapped partially with the above validated phenotype for activated CD8^+^ T cells (Fig. 6A, right). Furthermore, impaired organelle and lysosome homeostasis in aged CD4^+^ T cells has been previously described as a relevant process in aging of T cells (*57*).

Pathway enrichments for oxidative phosphorylation and mitochondrial respiration in age-associated T cell phenotypes were in line with reports of defective respiration in CD4^+^ T cells of aged mice (*58*, *59*) and suggested that the neural network might have identified a phenotypic T cell signature associated with both donor age and mitochondrial abundance. The CD4^+^ T cells showed strong bright-field intensity differences, a measure of intracellular granularity (Fig. 2, A and B, and fig. S5B). This bright-field trend followed the age associations that we observed, with CD4^+^ T cells enriched in younger people measured to be more granular (referred to as T4_BFD_ for “bright-field dark” CD4^+^ T cells; Figs. 2, A and B, and 4C). Quantifying this association across all subpopulations, CD4^+^ T cells showed the most significant age-associated bright-field intensity differences (*P* < 10^−70^), followed by the CD8^+^ T cells (*P* < 10^−40^), and less for the other immune cell classes (Fig. 6B).

To reproduce this association, we sampled an additional validation cohort of 15 healthy donors (Fig. 6C) and trained a different neural network architecture on a new set of images generated only from this validation cohort (fig. S6B). This independent repetition of the workflow revealed that the age-associated T4_BFD_ phenotype was independent of the donor cohort and neural network and experimental batch (Fig. 6C and fig. S6B). The age-associated bright-field intensity differences and mitochondrial pathway association might reflect loss of mitochondrial abundance in age in CD4^+^ T cells (*60*). To support this interpretation, we analyzed whether bright-field intensity reflects mitochondrial abundance using the natural heterogeneity observed within CD4^+^ T cells of a single donor (Fig. 6D). Those cells that were darkest by bright-field imaging displayed significantly higher mitochondrial abundance as measured by image-based quantification of the MitoTracker dye (Fig. 6D). The deep learning uncertainty thus had revealed a label-free phenotype reflecting an age-associated mitochondrial decline in CD4^+^ T cells, explaining, in part, how immune cell phenotypes measured by our high-throughput single-cell imaging pipeline capture donor information such as age.

## DISCUSSION

We here explore the molecular health determinants of human immune cell phenotypes using a workflow that combines automated high-throughput microscopy, single-round multiplexed immunofluorescence, and deep learning–based phenotypic analysis. The presented method for phenotyping of immune cells distinguishes itself for its ability to integrate cell morphology, protein levels and localization, and multicellular context into a quantitative metric across eight major immune cell classes, hundreds of conditions, and millions of cells. The resulting single-cell phenotype space, derived from the CNN’s uncertainty, reflected both genetic and nongenetic donor health information. We find age, gender, blood pressure, and inflammatory state to be significantly associated with human immune cell phenotypes, yet many more influences likely exist and more phenotype associations captured by our approach remain unexplored.

Our workflow is tailored to make use of two large sources of biological heterogeneity: the heterogeneity observed between individuals, and heterogeneity observed within cells of the same class and donor. That dependency, however, is at the same time its limitation: The single-round multiplexed staining strategy benefits from the presence of multiple cell types with variable cell morphologies and marker profiles, and LEA requires donor or condition heterogeneity to power its associations. Furthermore, while the marker panel shown here reliably captures the predominant immune cell classes present in PBMCs, it does not resolve certain smaller subpopulations, such as NK T cells (*61*). However, the approach is flexible as the panel composition can readily be tailored to the identification of additional subpopulations or adapted to different tissues, building on the same logic developed here.

This is not the first work that deploys CNN-based cell classification (*62*–*70*) and feature extraction (*63*, *65*, *69*, *71*). Here, we apply deep learning in high-throughput screening and phenotypic analyses of primary human PBMCs. By training the CNN on curated cells from across independent experiments, multiple donors, and conventional and multiplexed staining panels, we could prevent overfitting on phenotypes of single donors and technical bias stemming from experimental conditions. However, the CNN class probability space, which we here successfully use as a phenotype discovery tool, is sensitive to different phenotypes resulting from different experimental conditions. Hence, while CNN classification can be trained to be robust, experimental care needs to be taken when interpreting the CNN class probability space.

Once new phenotypes are found, as we demonstrate for the inflammation-associated T_ACT_ cell morphology, the ability to retroactively reclassify cells based on their morphology with dedicated CNNs allows robust morphological subclassification of previously imaged cells even in the absence of tailored marker panels. Attesting to the robustness of the found phenotypes, the inflammation-associated T_ACT_ and age-associated T4_BFD_ phenotypes could be validated in independent experiments, in an independent validation cohort, using distinct neural network architectures, and, for the T_ACT_ morphology, in both live-cell and fixed sample imaging.

In the future, repeated profiling of individual donors will allow to further stratify temporally stable from dynamic immune cell phenotypes. Furthermore, comparative studies across larger patient and donor cohorts, as well as identifying clinically relevant cell morphologies in the context of personalized treatment identification for hematological malignancies (*41*, *42*), will be additionally attractive avenues of study. This will inevitably define the boundaries of the personal health information reflected by immune cell phenotypes. Given that the workflow allows simultaneous phenotype discovery combined with the molecular and personal health associations, it is well positioned to lead to the discovery of more as yet undescribed and clinically relevant immune cell phenotypes.

## MATERIALS AND METHODS

### Experimental model

Buffy coats or whole blood tubes were obtained from coded healthy donors provided by the Blutspende Zürich, under a study protocol approved by the Cantonal Ethics Committee, Zürich (KEK Zürich, BASEC-Nr 2019-01579). Detailed donor information can be found in table S3.

### Experimental details

#### Collection and purification of human PBMCs

Healthy donor buffy coats or blood samples were diluted 1:1 in phosphate-buffered saline (PBS; Gibco), and PBMCs were isolated with a Histopaque-1077 density gradient (Sigma-Aldrich) according to the manufacturer’s instructions. PBMCs at the interface were collected, washed once in PBS, and resuspended in medium. In all experiments, immune cells were cultured in RPMI 1640 and GlutaMAX medium (Gibco) supplemented with 10% fetal bovine serum (FBS; Gibco) and incubated at 37°C with 5% CO_2_. Cell number and viability were determined using a Countess II Cell Counter from Thermo Fisher Scientific according to the manufacturer’s instructions.

#### Nonadherent PBMC monolayer formation and drug screening and cell fixation

In the proof-of-concept drug screen, 5 μl of a selected screening compounds (10× stock) and all respective controls (as outlined in table S2) were transferred to CellCarrier 384 Ultra, clear-bottom, tissue culture–treated plates (PerkinElmer) with five replicates per condition. All conditions were screened in four concentrations: cytokines (0.1, 1, 10, and 100 ng/ml), rituximab (0.05, 0.1, 0.5, and 1 μg/ml), LPS (0.1, 1, 10, and 100 ng/ml), dexamethasone (0.4, 4, 40, and 400 ng/ml), and crizotinib (0.01, 0.1, 1, and 10 μM). Fifty microliters of medium containing approximately 4 × 10^5^ cells/ml was pipetted into each well of a 384-well compound plate, and cells were allowed to settle to the bottom. The whole blood samples of the discovery cohort (shown in Figs. 2, A and B, and 4 to 6) were incubated for 1 hour, whereas all buffy coat samples, including all samples from the validation cohort (Figs. 5H and 6C), were incubated for 24 hours. All assays were terminated by fixing and permeabilizing the cells with 20 μl of a solution containing 0.5% (w/v) formaldehyde (Sigma-Aldrich), 0.05% (v/v) Triton X-100 (Sigma-Aldrich), 10 mM sodium(meta)periodate (Sigma-Aldrich), and 75 mM l-lysine monohydrochloride (Sigma-Aldrich), for 20 min at room temperature. For MitoTracker staining (Thermo Fisher Scientific), cells were stained live with 500 nM MitoTracker Red, before fixation. Fixative-containing medium was subsequently removed, and cells were blocked and photobleached in 5% FBS/PBS overnight at 4°C. Photobleaching was used to reduce background fluorescence and was performed by illuminating the fixed cells with conventional white light light-emitting diode panels.

#### Immunostaining and imaging

All fluorescent primary antibodies used in this work (outlined in table S1) were used at a 1:300 dilution in PBS. All antibody cocktails for immunohistochemistry (IHC) contained 6 μM DAPI (Sigma-Aldrich) for nuclear detection. Before IHC staining, the blocking solution was removed, and 20 μl of the antibody cocktail was added per well and incubated for 1 hour at room temperature. Besides fully multiplexed wells, each plate additionally contained several staining control wells with a reduced number of antibodies (table S1). The staining control wells served for evaluating antibody functionality and the generation of the CNN training data (see below). For imaging, a PerkinElmer Opera Phenix automated spinning disk confocal microscope was used. Each well of a 384-well plate was imaged at ×20 magnification with 5 × 5 nonoverlapping images, covering the whole well surface. The images were taken sequentially from the bright-field (650 to 760 nm), DAPI/nuclear signal (435 to 480 nm), green fluorescent protein/green signal (500 to 550 nm), phycoerythrin/orange signal (570 to 630 nm), and APC/red signal (650 to 760 nm) channels. Subsequently, the raw .tiff images were transferred from the microscope for further analysis.

#### Conventional image analysis and quality filtering

Cell detection and single-cell image analysis were performed using CellProfiler v2 (*72*). Nuclear segmentation was performed via thresholding on DAPI intensity. Cellular outlines were estimated by a circular expansion from the outlines of the nucleus. In addition, a second and larger expansion from the nuclei was performed to measure the local area around each single cell (local cellular background). Standard CellProfiler-based intensity, shape, and texture features of the nucleus and cytoplasm and the local cell proximity were extracted for each measured channel. Raw fluorescent intensities were log_10_-transformed and normalized toward the local cellular background as described by Vladimer *et al.* (*40*).

#### Convolutional neural networks

CNNs used in this work were implemented using MATLAB’s Neural Network Toolbox version R2020a. The curated dataset used in training, validation, and testing of the CNN framework contains images of cells from fully multiplexed stainings and images from staining controls. Staining controls were designed to contain only a subset of the antibodies used in the multiplexed setting (table S1). This reduced complexity first enables to evaluate the functionality of the selected antibody and the presence of the targeted antigen in each sample. Furthermore, antibody combinations in the staining controls were picked to mirror the staining of the selected subpopulation in the multiplexed setting (e.g., staining control 1 only contained antibodies marking T cell–specific antigens; T cells in the multiplexed setting will have the same staining pattern). The same staining patterns in the controls and the mostly nonoverlapping emission spectra of the chosen antibodies allow an easy, marker intensity–based identification of subpopulations. This facilitates a fast and unbiased selection of training examples. For the generation of single-cell images, the center of each cell was determined by its nuclear staining via the software CellProfiler (see above). Around each nuclei center, a 50-pixel by 50-pixel (or 39.5-μm by 39.5-μm) wide subimage was generated across all five measured channels. Single-cell subimages were then manually annotated and sorted for their respective class using custom MATLAB scripts. For training and validation of the discovery cohort CNN, a dataset of 89,483 cells was manually annotated (containing both multiplexed and control staining cells). In the separate test datasets, each donor-associated set is independently split in multiplexed and control staining cells, resulting in a total of 30 independent test datasets with each 100 cells per class. This test setup allows inferring the network performance toward each donor, experiment, and staining type independently.

In discovery cohort (10 donors), a 17-layer-deep CNN with an adapted “AlexNet” architecture (*73*) with 50-pixel by 50-pixel and five-channel input images was used. Before training, the labeled eight-class dataset was randomly split in a training set containing 90% and a validation set with the remaining 10% of all images. Network layer weights and biases were initialized randomly before the CNN network was trained. Networks were trained up to 20 epochs with a mini batch size of 512 images. The learning rate was fixed to 0.0001. To avoid overfitting, L2 regularization with 0.005 was applied. Furthermore, in each iteration, input images were randomly rotated in 45° steps with an additional possibility to be also flipped vertically or horizontally. Performance of the trained networked was tested on the separate test sets of staining control and multiplexed images of all 15 donors. Stochastic gradient descent with momentum of 0.9 is defined as the optimization algorithm. Last, we trained 20 differently initialized networks with differently split training and validation sets. For the final classification of the complete unlabeled dataset, the best performing network was used. As in the generation of the labeled dataset, 50-pixel by 50-pixel subimages around each nuclei center were generated. Cells closer than 25 pixels to the border of an image were excluded from classification.

In validation cohort (15 donors), a 71-layer-deep CNN with an adapted ResNet architecture (*74*) with 48-pixel by 48-pixel and five-channel input images was used. Before classification and training, all intensity values were first log_10_-transformed and then channel wise–normalized to a range of 0 to 1. The eight-class CNN was trained using randomly initialized weights and biases and the adaptive learning rate optimization “ADAM.” The network was trained for 20 epochs with an initial learning rate of 0.001, which was dropped every 5 epochs with a factor of 0.1. Furthermore, a mini batch size of 512 images and L2 regularization with 0.001 was applied. To further strengthen generalization, input images were augmented in each iteration. Here, images were randomly rotated in 45° steps with an additional possibility to be also flipped vertically or horizontally. To block an overreliance on absolute intensity values, channel intensity shifts were simulated via a multiplication with a random fixed factor. This used factor was randomly drawn out of a normal distribution with a mean of 1 and an SD of 0.2. Furthermore, images were augmented with random noise (specifically salt and pepper noise, speckle noise, Gaussian noise, or image blurring). In all CNN classifications, 48-pixel by 48-pixel subimages around each nuclei center were generated. Cells closer than 24 pixels to the border of an image were excluded from all classifications.

In label-free T_ACT_ classier, CNNs and single-cell images were generated as described above. The labeled training and validation dataset comprised a total of 8862 cells (1:2 T_ACT_:T_CON_ ratio). CNNs were trained with a mini-batch size of 200 images to a maximum of 100 epochs, which could be terminated if validation loss was greater than the previous smallest loss for five consecutive times. In addition, the images were randomly rotated by 45° and mirrored vertically or horizontally per iteration to limit orientation bias toward polarized T_ACT_ cells. The CNN performance was assessed by classifying 1107 test cells (1:2 T_ACT_:T_CON_ ratio) that had neither been used in CNN training nor in validation.

#### RNA sequencing

In T cell isolation and RNA extraction, T cells were isolated from fresh PBMCs directly after obtaining them via density centrifugation, as described above. Isolation was performed via a column-based extraction method with CD3 Microbeads as described in the manufacturer’s instructions (Miltenyi Biotec). RNA extraction of the isolated cells was performed with a Quick-RNA MiniPrep Kit by Zymo according to manufacturer’s instructions.

RNA-seq was performed by the Functional Genomics Center Zürich. Briefly, cDNA libraries were obtained according to protocols published by Picelli *et al.* (*75*). Illumina library was obtained via tagmentation using the Illumina Nextera Kit. All samples were sequenced in a single run on a NovaSeq 6000 (single read, 100 base pairs, depth of 20 Mio reads per sample).

In data processing and normalization, Illumina adapters, sequences of poor quality, as well as polyadenine (polyA) and polythymine (polyT) sequences were removed from the raw reads using TrimGalore v.0.6.0 with cutadapt v.2.0 before alignment. Reads were then aligned to the human reference genome GRCh38, v93 (Ensembl) using STAR v. 2.5.3a. Reads per gene were counted using the –quantMode GeneCounts flag in STAR. Gene counts below a threshold of 20 raw counts were filtered, and raw counts were normalized [DESeq2 (*76*)]. Only transcripts annotated as “protein coding” or “long noncoding RNA” were considered in the subsequent analysis.

### Statistical analysis

If not stated otherwise, all significance scores were calculated on the basis of a two-tailed Student’s *t* test with mean of 0. Asterisks in figures indicate significance per condition, compared with controls. *P* value less than 0.05 is flagged with one asterisk (*), *P* value less than 0.01 is flagged with two asterisks (**), and all *P* values with less than 0.001 are flagged with three asterisks (***).

For cell-cell interaction analysis, a simplified version of the interaction method by Vladimer *et al.* (*40*) was used. Here, cell-cell interaction analysis was conducted over all different image sites within the same well. Cells were scored as interacting if their nuclear centroids were within a Euclidean distance of 40 pixels. To calculate the interaction score of a cell with type A interacting with a cell of type B, we first calculated specific interactions and total interactions per well. We define specific interactions, as the total count of “B” cells within the defined radius around a cell of type “A.” Total interactions are considered as the total count of all interacting cells in that well. To calculate the final interaction score, specific interactions were divided by the product of (the fraction of type A cells of all cells) × (the fraction of type B cells of all cells) × total interactions. In contrast to the previously published method, this approach is simplified as the interactions scores are nondirected, which reduces the number of edges from 72 to 36. Mean interaction score over all replicates was calculated, log_2_-transformed, and normalized toward its respective control (see table S2).

All *t*-SNE visualizations were calculated on the –log_10_(class probability matrices). In the *t*-SNE calculation, a Mahalanobis distance metric, a perplexity of 30, and an exaggeration parameter of 4 were applied. To reduce the calculation time, the Barnes-Hut algorithm with a θ value of 0.5 was used.

To calculate whether a certain condition displays local enrichment in the eight-dimensional class probability space, we developed the KNN LEA by hypergeometric testing or rank-based correlation. Here, we probe the local neighborhood around each single cell, which is defined as the KNNs in the original CNN class probability space. For discrete variables (such as donor identity), we calculate the probability to randomly find at least *n* cells of condition *X* in a certain neighborhood using a hypergeometric cumulative distribution function. This takes into account the total number of cells in the probed neighborhood, the total number of cells in the tested class probability space, and the total number of cells of condition *X*. In case of continuous variables (such as donor age or gene transcript counts), the relative fraction of cells of each donor in the probed local neighborhood is calculated. The fractions are then correlated (Spearman’s rank correlation) with a continuous variable, and the significance of the correlation is calculated. In both cases, the enrichment probability is assigned to the center cell of the probed region, and the approach is iterated for each single cell in the selected *n*-dimensional space. If not stated otherwise, neighborhoods were defined as *k* = 400 nearest neighbors for Figs 2 to 4 and figs. S2 to S4 and as *k* = 200 for the T cell for Figs. 5 and 6 and figs. S5 and S6. In general, KNN-based algorithms, such as KNN-based classifiers, can overfit when *K* is set too small or underfit when *K* is set too big. As a general rule of thumb, the square root of the number of data points in the dataset is a reasonable starting value for *K*. Please see data file S1 for a full example workflow of LEA. *P* values were corrected for multiple testing, i.e., by the number of total cells (i.e., tests) in the analysis.

In pathway enrichment analysis, pathway annotations were obtained using the DAVID database (*77*). Gene enrichments per single cell were calculated via LEA (see above). To calculate pathway enrichments per single cell, the LEA gene enrichments of all genes belonging to a certain pathway annotation were compared against the enrichment of all other genes. Significance scores were calculated on the basis of a two-tailed Student’s *t* test, and directionality was calculated by the difference of the means of both populations.

## Notes

View/request a protocol for this paper from *Bio-protocol*.
