# Supplementary material for: Multiplexed high-throughput immune cell imaging reveals molecular health-associated phenotypes
Source: Sci Adv. 2022 Nov 2;8(44):eabn5631. doi: 10.1126/sciadv.abn5631 (PMC9629716; doi:10.1126/sciadv.abn5631)
Supplement: Supplementary file 1 — Figs. S1 to S6 Tables S1 to S3 [file sciadv.abn5631_sm.pdf]

Science Advances
AAAS

Supplementary Materials for

Multiplexed high-throughput immune cell imaging reveals molecular health-associated phenotypes

Yannik Severin *et al.*

Corresponding author: Berend Snijder, snijder@imsb.biol.ethz.ch

Sci. Adv. **8**, eabn5631 (2022)
DOI: 10.1126/sciadv.abn5631

The PDF file includes:

Figs. S1 to S6
Legend for data file S1
Tables S1 to S3

Other Supplementary Material for this manuscript includes the following:

Data file S1

Fig. S1

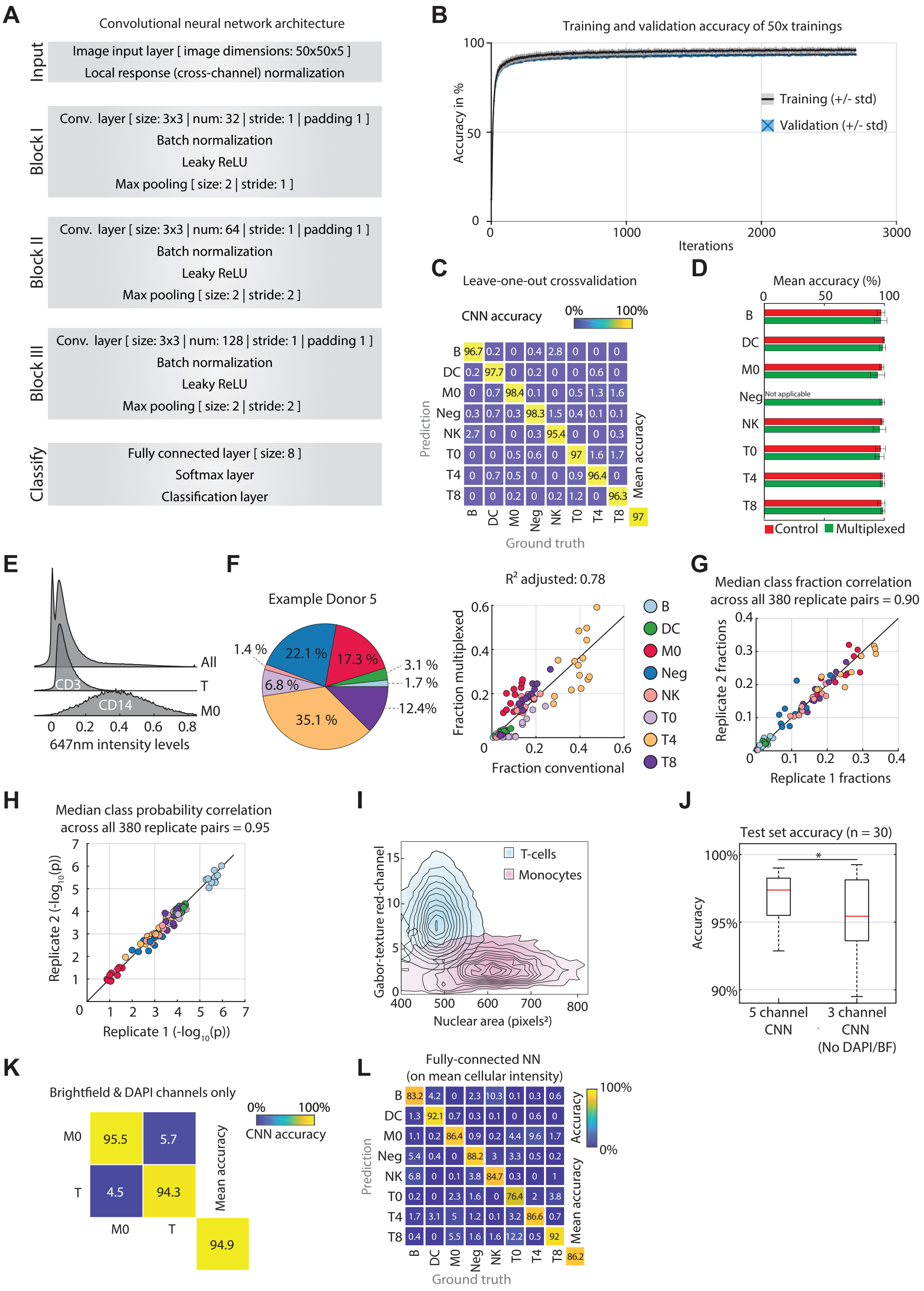

A
Convolutional neural network architecture
Input
Image input layer [image dimensions: 50x50x5]
Local response (cross-channel) normalization
Block I
Conv. layer [size: 3x3 | num: 32 | stride: 1 | padding 1]
Batch normalization
Leaky ReLU
Max pooling [size: 2 | stride: 1]
Block II
Conv. layer [size: 3x3 | num: 64 | stride: 1 | padding 1]
Batch normalization
Leaky ReLU
Max pooling [size: 2 | stride: 2]
Block III
Conv. layer [size: 3x3 | num: 128 | stride: 1 | padding 1]
Batch normalization
Leaky ReLU
Max pooling [size: 2 | stride: 2]
Classify
Fully connected layer [size: 8]
Softmax layer
Classification layer
B
Training and validation accuracy of 50x trainings
Accuracy in %
Iterations
Training (+/- std)
Validation (+/- std)
C
Leave-one-out crossvalidation
CNN accuracy
0%
100%
Prediction
Ground truth
Mean accuracy
B 96.7 0.2 0 0.4 2.8 0 0 0
DC 0.2 97.7 0 0.2 0 0 0.6 0
M0 0 0.7 98.4 0.1 0 0.5 1.3 1.6
Neg 0.3 0.7 0.3 98.3 1.5 0.4 0.1 0.1
NK 2.7 0 0 0.3 95.4 0 0 0.3
T0 0 0 0.5 0.6 0 97 1.6 1.7
T4 0 0.7 0.5 0 0 0.9 96.4 0
T8 0 0 0.2 0 0.2 1.2 0 96.3
B DC M0 Neg NK T0 T4 T8
97
D
Mean accuracy (%)
B
DC
M0
Neg
Not applicable
NK
T0
T4
T8
Control
Multiplexed
E
All
T
M0
CD3
CD14
647nm intensity levels
F
Example Donor 5
22.1 %
17.3 %
1.4 %
6.8 %
35.1 %
3.1 %
1.7 %
12.4%
R² adjusted: 0.78
Fraction multiplexed
Fraction conventional
B
DC
M0
Neg
NK
T0
T4
T8
G
Median class fraction correlation
across all 380 replicate pairs = 0.90
Replicate 2 fractions
Replicate 1 fractions
H
Median class probability correlation
across all 380 replicate pairs = 0.95
Replicate 2 (-log10(p))
Replicate 1 (-log10(p))
I
T-cells
Monocytes
Gabor-texture red-channel
Nuclear area (pixels²)
J
Test set accuracy (n = 30)
Accuracy
100%
95%
90%
*
5 channel CNN
3 channel CNN (No DAPI/BF)
K
Brightfield & DAPI channels only
0%
100%
CNN accuracy
M0 95.5 5.7
T 4.5 94.3
M0 T
Mean accuracy
94.9
L
Fully-connected NN
(on mean cellular intensity)
Prediction
Ground truth
Accuracy
Mean accuracy
100%
0%
B 83.2 4.2 0 2.3 10.3 0.1 0.3 0.6
DC 1.3 92.1 0.7 0.3 0.1 0 0.6 0
M0 1.1 0.2 86.4 0.9 0.2 4.4 9.6 1.7
Neg 5.4 0.4 0 88.2 3 3.3 0.5 0.2
NK 6.8 0 0.1 3.8 84.7 0.3 0 1
T0 0.2 0 2.3 1.6 0 76.4 2 3.8
T4 1.7 3.1 5 1.2 0.1 3.2 86.6 0.7
T8 0.4 0 5.5 1.6 1.6 12.2 0.5 92
B DC M0 Neg NK T0 T4 T8
86.2

Figure S1. Training and validation of the deep learning-enabled multiplexed PBMC imaging workflow

(A) Overview of the convolutional neural network architecture. **(B)** Average and standard deviation of training and validation accuracies over 20 randomly initialized CNN instances. Validation set represents 10% of the initial training set (n=8948). Network training after 20 epoches. **(C)** Confusion matrix of CNN performance on a leave-one-out cross validation per donor. The CNN was trained with 14 donors and subsequently tested on an unseen donor not included in the training dataset. The confusion matrix shows the mean accuracies after iterating across all donors. **(D)** Comparison of prediction accuracy on conventionally stained and multiplexed cells. Bar plots show the mean accuracy (in %) and the standard deviation of the CNN prediction across all donors individually per class and staining. **(E)** Distribution of 647nm intensity levels across all cells (upper), classified $CD3^+$ T-cells (middle) and classified $CD14^+$ monocytes (lowest) of Donor 1. A cell class probability threshold of 0.8 was applied. **(F)** Population percentages for Donor 5 (left) and class fraction comparison of conventionally stained and multiplexed cells across 15 healthy donors. Negative cell class is excluded due to its unavailability in conventional stainings. **(G)** Class fraction comparison of two single replicates (plate wells) across all 10 donors. Each dot corresponds to a replicate pair from a single donor. Color indicates the cell type. The median pairwise correlation across all technical replicates is indicated. **(H)** Median class probability comparison of two single replicates (plate wells) across all 15 donors. Shown statistic depicts the median class probability correlation of all pairwise replicate combinations per donor across two individual 384 well plates. **(I)** Comparison of selected morphological and staining-pattern parameters divergent between T-cells and monocytes. Conventionally stained T-cells and monocytes from Donor 1 were identified by immunofluorescence gating for CD3 and CD14, respectively. Morphological features were extracted by CellProfiler. **(J)** Test set accuracies of either a CNN trained on all 5 channels, or trained without the DAPI & BF channel. Boxplots show the accuracies of the multiplexed and control test-sets of each of the 15 donors; n=30. **(K)** Confusion matrix of CNN performance on classifying T-cells and monocytes based on brightfield and DAPI channels only. An adapted CNN architecture (2-channel input and 2 class output) was trained with 1900 2-channel images of T-cells and monocytes. Network performance was evaluated in the curated test set containing 750 cells per class. **(L)** Confusion matrix of the performance of a feedforward fully-connected neural network with a size of 10 nodes trained on mean channel intensity measurements. Mean intensity features per single-cell were measured using CellProfiler for all five channels, matching the cells used in the CNN training and test sets.

Fig. S2

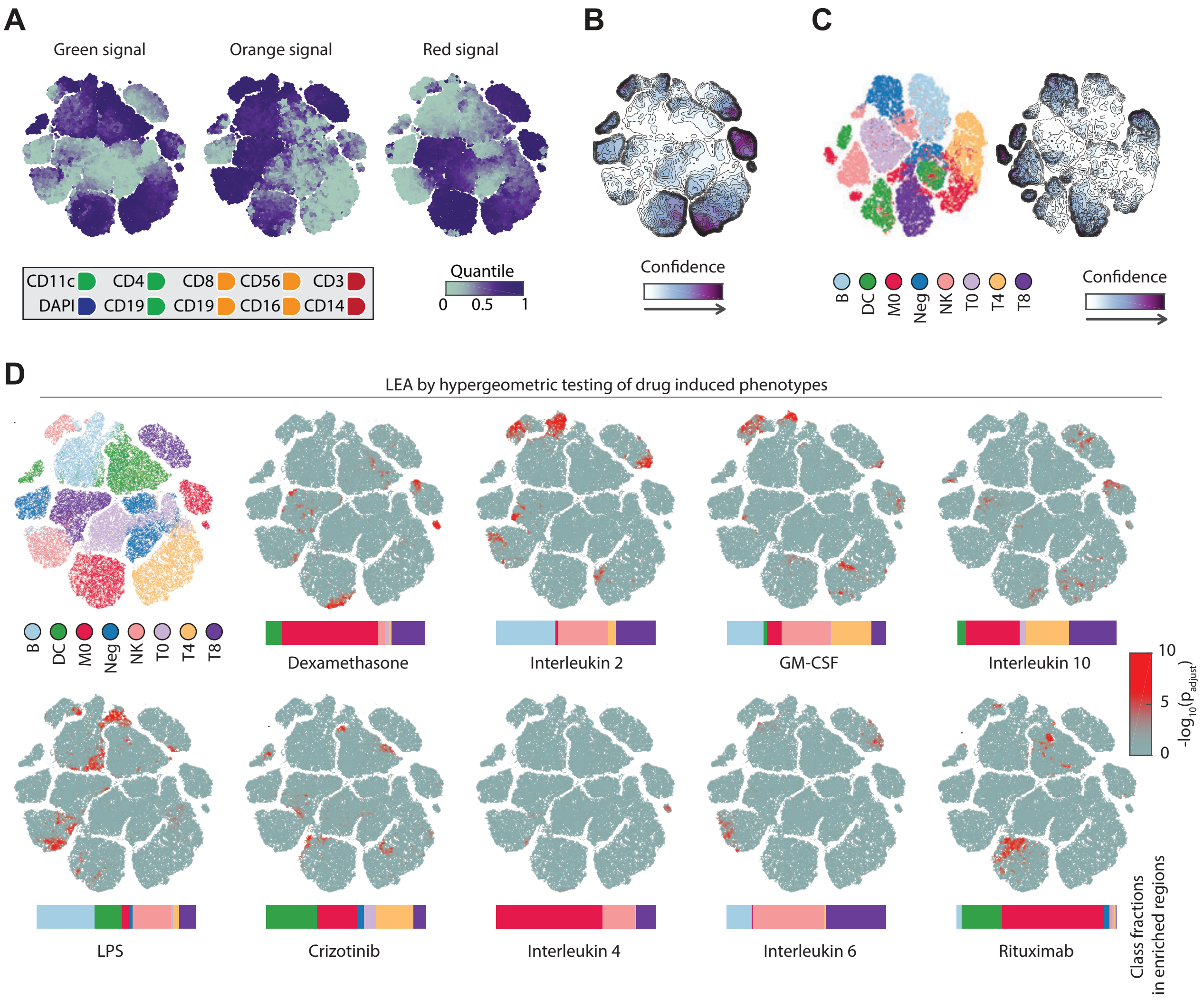
A
Green signal
Orange signal
Red signal
CD11c CD4 CD8 CD56 CD3
DAPI CD19 CD19 CD16 CD14
Quantile
0 0.5 1
B
Confidence
C
B DC M0 Neg NK T0 T4 T8
Confidence
D
LEA by hypergeometric testing of drug induced phenotypes
B DC M0 Neg NK T0 T4 T8
Dexamethasone
Interleukin 2
GM-CSF
Interleukin 10
10
5
0
$-\log_{10}(p_{adjust})$
LPS
Crizotinib
Interleukin 4
Interleukin 6
Rituximab
Class fractions in enriched regions

Figure S2. Detailed view on the perturbed immune system of a single donor.

(A) Selected single-cell features mapped onto the same t-SNE map as depicted in Figure 3A. Median value of overlapping data points is calculated and color is assigned accordingly. Points are plotted in order of intensity, from highest to lowest. **(B)** Associated CNN probability contour plot of the phenotypic landscape of the immune system depicted in Figure 3A. **(C)** Left: Phenotypic landscape of the immune system across ten healthy donors. t-SNE embedding of the 8-class CNN probabilities without a confidence threshold of up to 1000 randomly subsampled multiplexed cells per class and per donor. Right: Associated CNN probability contour plot of the phenotypic landscape depicted left. **(D)** LEAs visualized by t-SNE of drug induced phenotypes. Horizontal bar graphs indicate the class fractions in enriched regions (at $p_{adjust} < 0.01$).

Fig. S3

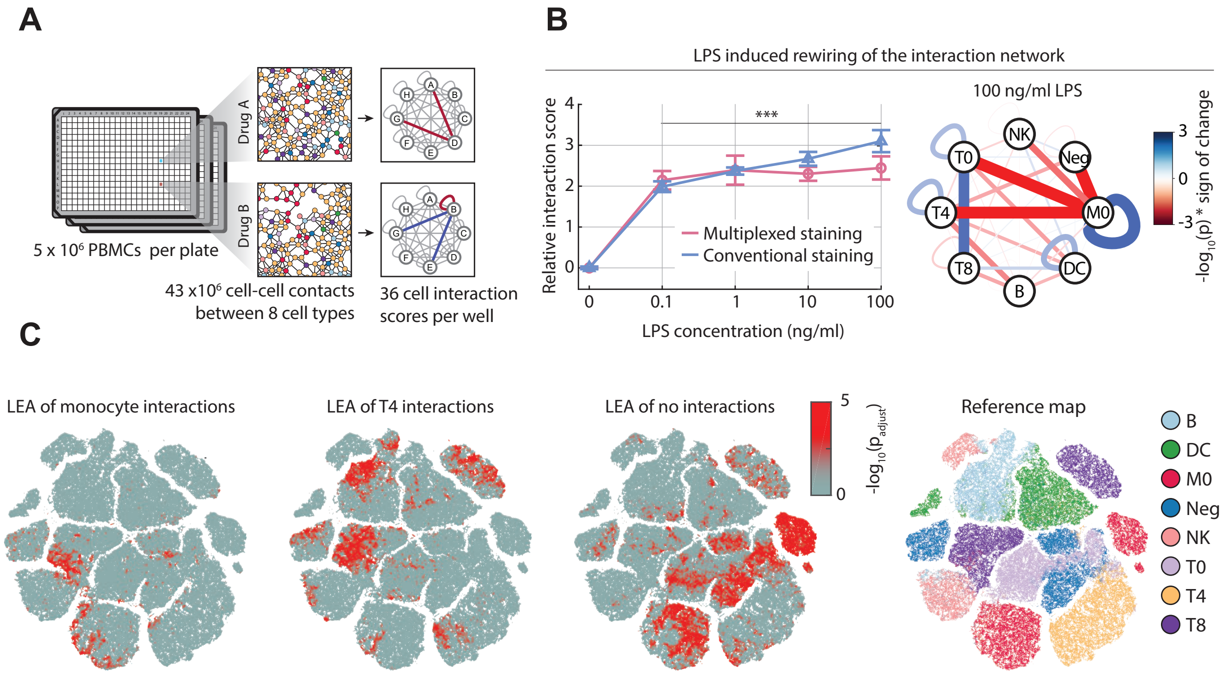
A
Drug A
Drug B
5 x 10⁶ PBMCs per plate
43 x10⁶ cell-cell contacts between 8 cell types
36 cell interaction scores per well
B
LPS induced rewiring of the interaction network
Relative interaction score
***
Multiplexed staining
Conventional staining
LPS concentration (ng/ml)
100 ng/ml LPS
NK
T0
Neg
T4
M0
T8
DC
B
-log10(p) * sign of change
C
LEA of monocyte interactions
LEA of T4 interactions
LEA of no interactions
-log10(padjust)
Reference map
B
DC
M0
Neg
NK
T0
T4
T8

Figure S3. Perturbation-induced rewiring of cell-cell contacts of the human immune system.

(A) Overview of cell-cell contact analysis over five million PBMCs. Cell-to-cell interaction networks between eight different immune cell populations with a total of 36 cell type interactions were generated per well, and compared across conditions. **(B)** LPS-induced rewiring of the cell-to-cell interaction network. Relative monocyte-to-monocyte interaction scores of multiplexed and conventionally stained wells as a function of increasing LPS concentration (left). Mean interaction score across all replicates is calculated and normalized against control treatment. Example LPS interaction network for 100ng/ml LPS (right). Significance of interaction ($-\log_{10}(p)$, multiplied times the sign of the phenotype (either positive or negative interaction score)). **(C)** LEA of cells with monocytes (left), T-cells (middle) or no-nearest neighbor (right).

Fig. S4

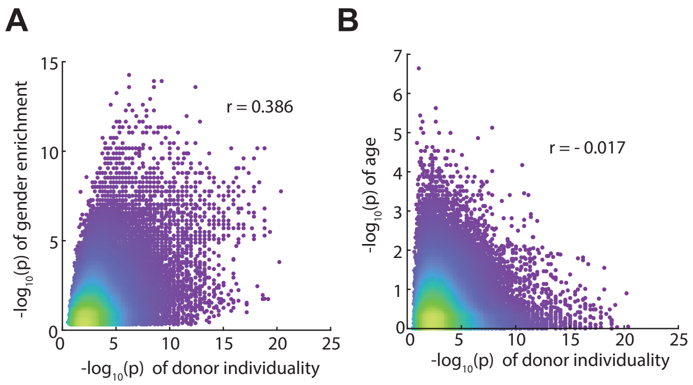
A
B
r = 0.386
r = - 0.017
-log10(p) of gender enrichment
-log10(p) of age
-log10(p) of donor individuality
-log10(p) of donor individuality

Figure S4. Comparative analysis of LEA-based phenotype and health parameter associations

(A) Comparison of donor LEA enrichments (as in Figure 4A) vs gender LEA enrichments (as in Figure 4B) per single cell. r values represent Pearson correlations. **(B)** Comparison of donor LEA enrichments vs age LEA enrichments per single cell (as in Figure 4D). R values represent pearson correlations.

Fig. S5

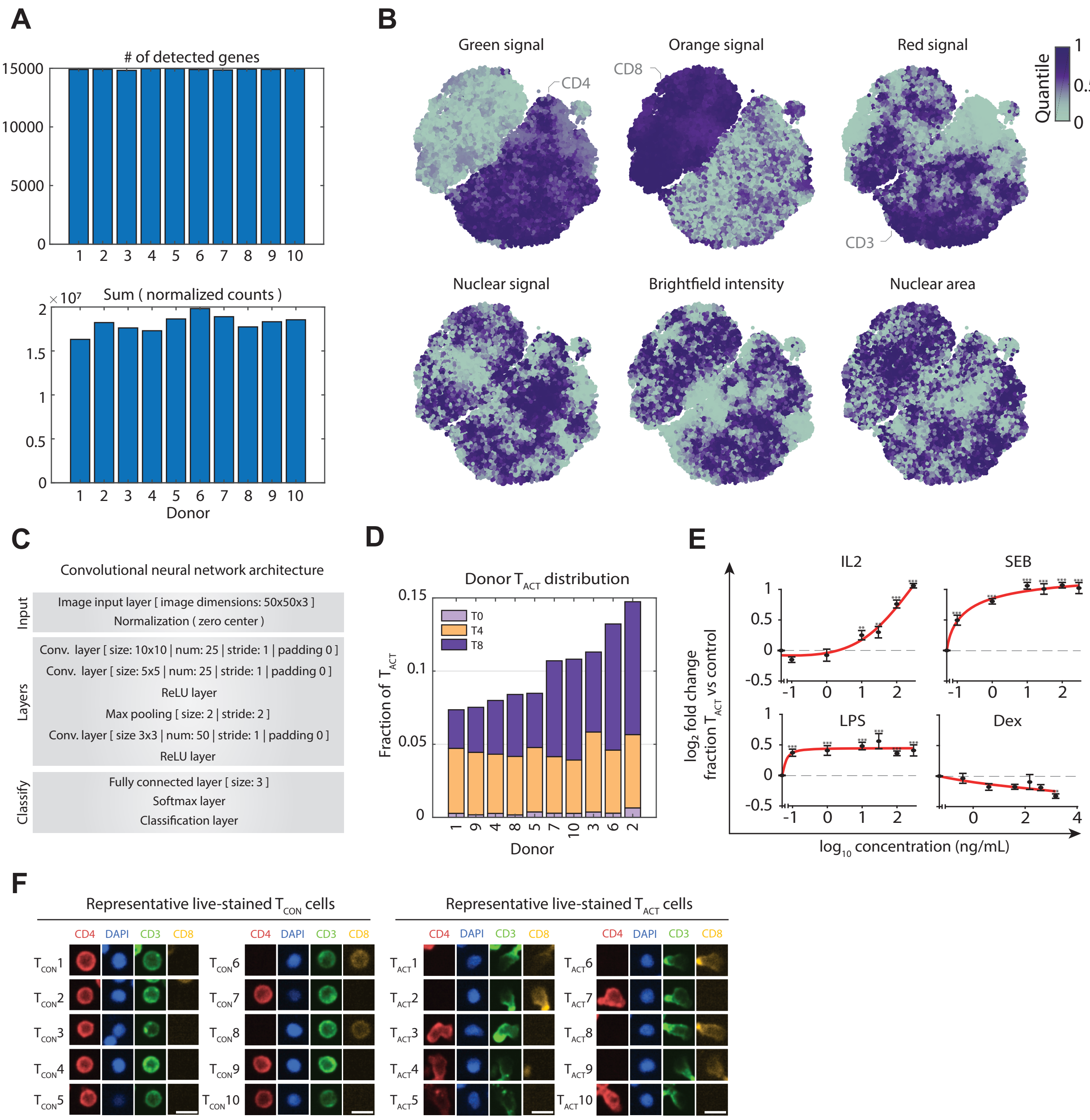

A
# of detected genes
Sum (normalized counts)
Donor
B
Green signal
Orange signal
Red signal
CD4
CD8
CD3
Quantile
Nuclear signal
Brightfield intensity
Nuclear area
C
Convolutional neural network architecture
Input
Image input layer [image dimensions: 50x50x3]
Normalization (zero center)
Layers
Conv. layer [size: 10x10 | num: 25 | stride: 1 | padding 0]
Conv. layer [size: 5x5 | num: 25 | stride: 1 | padding 0]
ReLU layer
Max pooling [size: 2 | stride: 2]
Conv. layer [size 3x3 | num: 50 | stride: 1 | padding 0]
ReLU layer
Classify
Fully connected layer [size: 3]
Softmax layer
Classification layer
D
Donor T_ACT distribution
Fraction of T_ACT
T0
T4
T8
Donor
E
IL2
SEB
LPS
Dex
log2 fold change fraction T_ACT vs control
log10 concentration (ng/mL)
F
Representative live-stained T_CON cells
Representative live-stained T_ACT cells
CD4
DAPI
CD3
CD8

Figure S5. T-cell transcriptomics, phenotyping, and validation of an activated T-cell morphology.

(A) Upper: Bar graphs indicate the number of detected transcripts (protein coding and long non-coding RNAs) after applying a threshold of 20 raw counts. Lower: Bar graphs indicated the sum of transcript counts after DESeq2 normalization (*76*). **(B)** Selected single-cell features projected onto the t-SNE depicted in Figure 5A. Median value of overlapping data points is calculated and color is assigned accordingly. Points are plotted in ascending order with the lowest intensity on top. **(C)** Overview of the label-free T-cell activation (T_{ACT}) convolutional neural network architecture. **(D)** Fraction of T_{ACT} cells per class and per donor. Stacked bar plots show the mean fraction of all T-cells per donor classified as T_{ACT}, within their respective T-cell subclass (T0, T4 or T8) in control (DMSO) conditions. **(E)** Induction and suppression of the T_{ACT} cell phenotype by immunomodulatory agents. Plotted are the $\log_2$ fold changes of the mean fraction of T-cells classified as T_{ACT} across all wells of each drug condition compared to control treatments. Cells were incubated with immunomodulatory agents at 0.1, 1, 10, 30, 100 and 300 ng/ml. Error bars show the standard error of the mean across wells for each drug condition. A custom Hill function (adjusted to different minima and maxima) was used to fit the data (red line). **(E)** Representative live-stained T_{ACT} and T_{CON} cell morphologies. Crop-size is 15 x 15 µm. All scale bars = 10µm.

Fig. S6

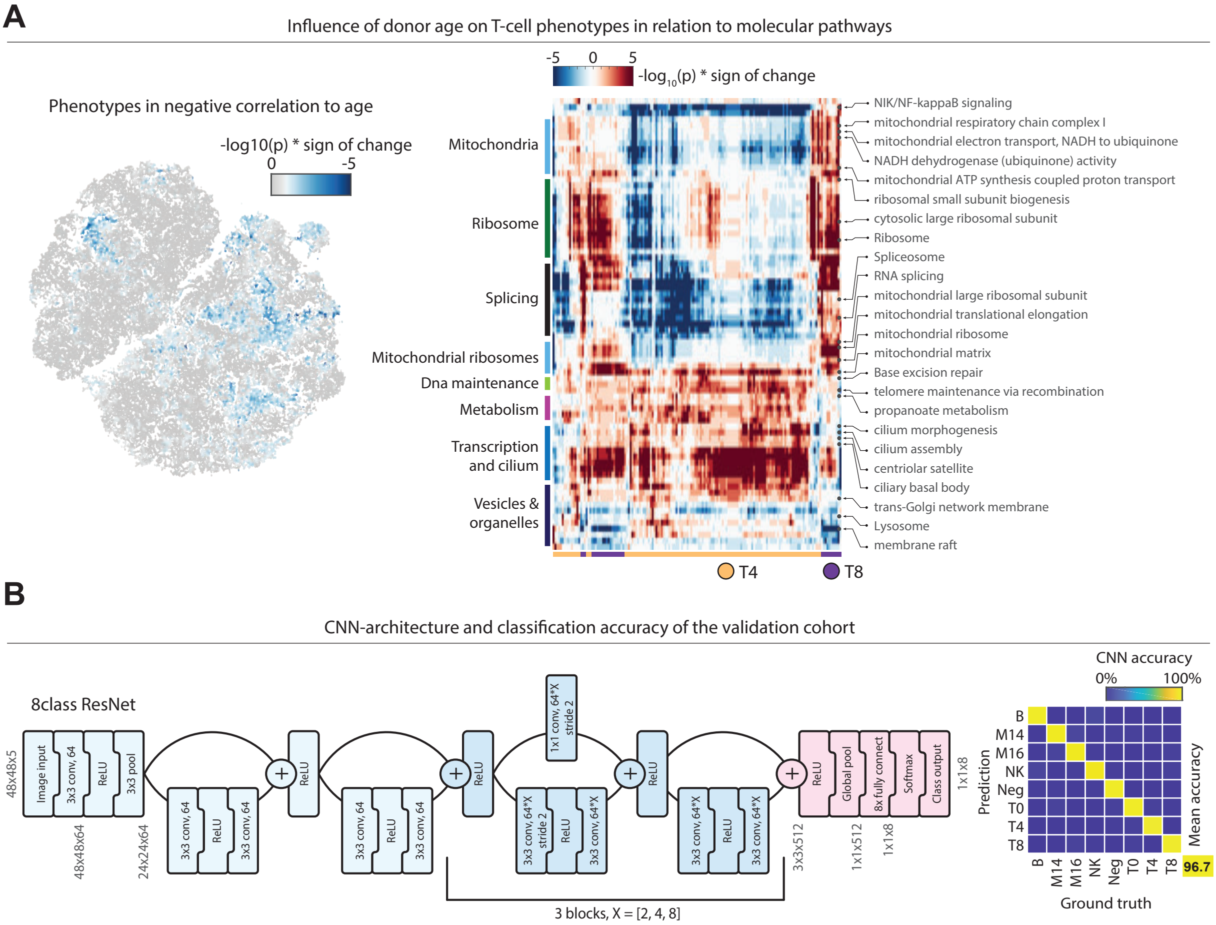

A
Influence of donor age on T-cell phenotypes in relation to molecular pathways
Phenotypes in negative correlation to age
-log10(p) * sign of change
0
-5
-5
0
5
-log10(p) * sign of change
Mitochondria
Ribosome
Splicing
Mitochondrial ribosomes
Dna maintenance
Metabolism
Transcription and cilium
Vesicles & organelles
NIK/NF-kappaB signaling
mitochondrial respiratory chain complex I
mitochondrial electron transport, NADH to ubiquinone
NADH dehydrogenase (ubiquinone) activity
mitochondrial ATP synthesis coupled proton transport
ribosomal small subunit biogenesis
cytosolic large ribosomal subunit
Ribosome
Spliceosome
RNA splicing
mitochondrial large ribosomal subunit
mitochondrial translational elongation
mitochondrial ribosome
mitochondrial matrix
Base excision repair
telomere maintenance via recombination
propanoate metabolism
cilium morphogenesis
cilium assembly
centriolar satellite
ciliary basal body
trans-Golgi network membrane
Lysosome
membrane raft
T4
T8
B
CNN-architecture and classification accuracy of the validation cohort
8class ResNet
48x48x5
Image input
3x3 conv, 64
ReLU
3x3 pool
48x48x64
24x24x64
3x3 conv, 64
ReLU
3x3 conv, 64
ReLU
3x3 conv, 64
ReLU
3x3 conv, 64
ReLU
1x1 conv, 64*X stride 2
3x3 conv, 64*X stride 2
ReLU
3x3 conv, 64*X
ReLU
3x3 conv, 64*X
ReLU
3x3 conv, 64*X
3x3x512
ReLU
Global pool
8x fully connect
Softmax
Class output
1x1x512
1x1x8
1x1x8
3 blocks, X = [2, 4, 8]
CNN accuracy
0%
100%
B
M14
M16
NK
Neg
T0
T4
T8
Prediction
Ground truth
Mean accuracy
96.7

Figure S6. Influence of donor age on T-cell phenotypes in relation to molecular pathways.

(A) Left: negative LEA associations with donor age projected onto the t-SNE (colored by $-\log_{10}(p)$). Right: Heatmap overview of all significantly enriched pathways in positive age-associated T-cells ($-\log_{10}(p)<-5$). Rows are annotations, columns are significantly age-associated cells. **(B)** Left: Schematic of the 8-class ResNet architecture used for the 15 donor validation cohort. Right: Confusion matrix of the CNN. CNN was tested on 1000 cells per class that the CNN did not see before.

Supplementary Data File 1. Example LEA implementation.

This zip file contains an example MATLAB implementation of a LEA analysis. The data file contains the LEA code, an example script with implementation, and the data corresponding to Figure 3 and S2, including all relevant and required annotations. The code has been tested on MATLAB version R2020b.

Supplementary Table 1

Epitope	Vendour	Fluorophore	Clone	Host	Staining Control	Used in muliplexed	Lot
CD3	BioLegend	Alexa Fluor 647	UCHT1	Mouse	1	yes	B246715
CD4	BioLegend	FITC	SK3	Mouse	1	yes	B244280
CD8	BioLegend	Alexa Fluor 594	RPA-T8	Mouse	1	yes	B200099
CD19	BioLegend	FITC	SJ25C1	Mouse	3	yes	B239447
CD19	BioLegend	PE	SJ25C1	Mouse	3	yes	B237928
CD56	Beckman Coulter	PE	N901	Mouse	2	yes	52
CD16	BioLegend	PE	3G8	Mouse	2	yes	B238510
CD14	BioLegend	Alexa Fluor 647	HCD14	Mouse	2&3	yes	B260484
CD11c	BioLegend	Alexa Fluor 488	3.9	Mouse	2&3	yes	B209841
CD3	BioLegend	Alexa Fluor 488	UCHT1	Mouse	/	no	B278994
CD4	Biolegend	Alexa Fluor 647	SK3	Mouse	/	no	B293054
CD8	Biolegend	Alexa Fluor 594	RPA-T8	Mouse	/	no	B200099
pNFkB p65 (Ser529)	eBioscience	PE	B33B4WP	Mouse	/	no	4303324
pERK1/2 (Thr202 Tyr204)	Thermo Fisher Scientific	PE	MILAN8R	Mouse	/	no	4337535

Supplementary Table 1. Staining panel details.

Supplementary Table 2

Compound name	Vendour	Assay conc.	Carrier solution and control
Crizotinib	Sigma-Aldrich	10uM	1% DMSO
		1uM	0.1% DMSO
		0.1uM	0.01% DMSO
		0.01uM	0.001% DMSO
Dexamethasone	Sigma-Aldrich	400ng/ml	1% DMSO
		40ng/ml	0.1% DMSO
		4ng/ml	0.01% DMSO
		0.4ng/ml	0.001% DMSO
Lipopolysaccharide from Escherichia coli	Sigma-Aldrich	100ng/ml	PBS
		10ng/ml	PBS
		1ng/ml	PBS
		0.1ng/ml	PBS
Rituximab	Absolute antibody	1ug/ml	PBS
		0.5ug/ml	PBS
		0.1ug/ml	PBS
		0.05ug/ml	PBS
Recombinant Human IL-2	PeproTech	100ng/ml	0.001% (w/v) BSA in PBS
		10ng/ml	0.0001% (w/v) BSA in PBS
		1ng/ml	0.00001% (w/v) BSA in PBS
		0.1ng/ml	0.000001% (w/v) BSA in PBS
Recombinant Human IL-4	PeproTech	100ng/ml	0.001% (w/v) BSA in PBS
		10ng/ml	0.0001% (w/v) BSA in PBS
		1ng/ml	0.00001% (w/v) BSA in PBS
		0.1ng/ml	0.000001% (w/v) BSA in PBS
Recombinant Human IL-6	PeproTech	100ng/ml	0.001% (w/v) BSA in PBS
		10ng/ml	0.0001% (w/v) BSA in PBS
		1ng/ml	0.00001% (w/v) BSA in PBS
		0.1ng/ml	0.000001% (w/v) BSA in PBS
Recombinant Human IL-10	PeproTech	100ng/ml	0.001% (w/v) BSA in PBS
		10ng/ml	0.0001% (w/v) BSA in PBS
		1ng/ml	0.00001% (w/v) BSA in PBS
		0.1ng/ml	0.000001% (w/v) BSA in PBS
Recombinant Human G-MCSF	PeproTech	100ng/ml	0.001% (w/v) BSA in PBS
		10ng/ml	0.0001% (w/v) BSA in PBS
		1ng/ml	0.00001% (w/v) BSA in PBS
		0.1ng/ml	0.000001% (w/v) BSA in PBS

Supplementary Table 2. Immunomodulatory perturbations.

Supplementary Table 3

Discovery cohort (Figure 1, Figure 2, Figure 4, Figure 5 A-G, Figure 6 A-B)

Whole Blood Donor	Year of birth	Blood type	weight in kg	Height in cm	Gender	Blood pressure	Hb level
1	1999	A+	68	174	m	128/70	156
2	1987	A+	66	170	m	173/99	147
3	1990	A+	50	163	f	114/80	148
4	2000	A+	63	168	f	122/74	133
5	1968	0-	58	165	f	158/90	151
6	1974	B+	95	176	m	156/98	177
7	1950	0+	72	175	f	126/78	130
8	1967	A+	95	178	m	146/86	161
9	1967	0+	80	180	m	160/106	167
10	1965	A+	80	180	m	130/82	167

Immuno-modulatory screen (Figure 3)

Buffy coat donor	Year of birth	Blood type	weight in kg	Height in cm	Gender	Blood pressure	Hb level
1	1968	AB+	73	180	m	146/92	153

Validation cohort (Figure 5H, Figure 6C)

Buffy coat Donor	Year of birth	Blood type	weight in kg	Height in cm	Gender	Blood pressure	Hb level
1	1994	B+	65	183	m	135/77	152
2	1970	B+	69	177	f	148/92	147
3	1996	B+	75	192	m	138/91	157
4	1994	B+	85	180	m	124/74	159
5	1953	B-	65	178	m	142/95	168
6	1971	AB-	65	171	f	114/72	141
7	1991	B+	67	180	f	135/94	145
8	1963	B+	65	157	f	146/99	157
9	1963	B+	\	\	m	170/90	169
10	1953	0-	76	158	f	142/100	149
11	1999	AB+	61	169	f	131/74	136
12	1960	B+	90	182	m	155/98	151
13	1999	B+	66	173	m	171/103	157
14	1967	B+	80	182	m	162/102	174
15	1989	AB-	72	178	f	108/63	138

Supplementary Table 3. Overview of the human healthy donor cohorts.
